# 

**Published:** 1881-01

**Authors:** 


HERMAN MYNTER, M. D.

### CONTENTS:

PAGE.

ORIGINAL COMMUNICATIONS:
Fatty Heart. By H. R. Hopkins, M. D., . 241

CLINICAL REPORTS:
A Case of Traumatic Tetanus. Reported by J. W. Keene, M. D., . . . . . . 255
A Successful Case of Direct Transfusion for Pernicious Anæmia. Reported by Charles Cary, M. D., . . . . . . . 259

TRANSLATIONS:
Gunshot Wound of the Crural Artery and Vein, above the Profunda Artery and Vein. From the German by Herman Mynter, M. D., . . . . . . . . . 263

SELECTIONS:
On a Neglected Symptom in Breast-Cancer. By Herbert L. Snow, M. D., . . . . 268
A Positive Sign of Pregnancy during the First Three Months. By J. H. Carstens, M. D., . . . . . . . . 271
A Case of Superfœtation. By D. A. Walden, M. D., . . . . . . . . 273
Antiseptic Transfusion of Human Blood in a Patient the subject of Secondary Hemorrhage—Cure. By Wm. MacEwen, M.D., 273
Case of Intestinal Ohstruction or Occlusion, Lasting Thirty-Nine Days. By W. H. Lambart, M. D , T. C. D., Etc., . . . 276
Infantile Constipation, . . . . . . 280
Employment of a Milk Diet in Diseases of the Heart, . . . . . . . 281

EDITORIAL:
Enforcement of the Medical Law, . . 282
Spread of Scarlatina and Diphtheria, . . 283
Municipal Appointments Health Department 285
The Erie County Medical Society, . . 286
An Excellent Appointment, . . . . . 286

REVIEWS, . . . . . . 287

